# Electrocardiogram properties and risk of covert brain infarction and other magnetic resonance imaging abnormalities in a stroke‐free population

**DOI:** 10.1002/brb3.2991

**Published:** 2023-04-16

**Authors:** Fan Gao, Chen Chen, Fude Liu, Jianfeng Han

**Affiliations:** ^1^ Clinical Research Center The First Affiliated Hospital of Xi'an Jiaotong University Xi'an Shaanxi China; ^2^ Department of Neurology The First Affiliated Hospital of Xi'an Jiaotong University Xi'an Shaanxi China

**Keywords:** brain atrophy, brain infarction, electrocardiogram, white matter hyperintensity

## Abstract

**Objectives:**

This study aimed to investigate the association between electrocardiogram (ECG) abnormalities and silent vascular brain injury as defined by cerebral magnetic resonance imaging (MRI) in a stroke‐free community‐based population.

**Methods:**

A total of 5888 participants were studied from the Cardiovascular Health Study (CHS), a prospective cohort of community‐living older adults. Standard 12‐lead ECGs measured prior to MRI scan were used. MRI scans were conducted at years 4–6 and 10–11. The primary outcome was presence of incident covert brain infarcts (CBIs) on the 2nd MRI examination, excluding previous CBIs and stroke occurrence. Secondary outcomes included white matter, ventricular, and sulcal atrophy on the 1st MRI. Logistic and multiple linear regression models were used to assess the relationship between ECG findings and silent vascular brain injury.

**Results:**

Left axis deviation before MRI scan was related to presence of incident CBIs (odds ratio [OR]: 1.45; 95% CI: 1.01–2.08, *p* = .047). A long QT interval was associated with severe white matter hyperintensity (OR: 1.36; 95% CI: 1.04–1.77, *p* = .024). Minor Q and QS waves with ST‐T abnormalities were positively related to sulcal atrophy (*β*: 0.43, 95% CI: 0.06–0.81, *p* = .023).

**Conclusions:**

Our study found that ECG abnormalities were related to presence of CBIs, white matter hyperintensity, and sulcal atrophy on MRI in a stroke‐free relderly population. Specifically, those with left axis deviation had an increased risk of presence of CBIs.

## Graphical abstract

Our study found that electrocardiogram abnormalities were related to presence of covert brain infarcts (CBIs), white matter hyperintensity, and sulcal atrophy on MRI in a stroke‐free elderly population. Specifically, those with left axis deviation had an increased risk of presence of CBIs.Our findings suggest a potential important pathophysiologic role of heart electrical activity in the burden of silent vascular brain injury.

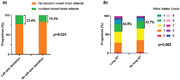

## 1 INTRODUCTION

Silent vascular brain injury occurs at an accelerated rate in elderly people and may cause subsequent overt clinical events, including stroke, vascular cognitive impairment, and death (Appleton et al., 2020; Cannistraro et al., 2019; Kalani et al., 2020; Leung et al., 2017). Subclinical vascular brain injury can be detected using cerebral magnetic resonance imaging (MRI), including white matter hyperintensity, silent infarcts, and gray matter atrophic changes (Appleton et al., 2020; Luoto et al., 2000). It has been reported that the prevalence of covert brain infarct (CBI) in people over 50 years old is higher (20%) than that of overt ischemic stroke (2%−14%) in the United States (Leung et al., 2017). Despite its high prevalence, an understanding of the pathogenesis and biomarkers of silent vascular brain injury is still developing (Cannistraro et al., 2019). In addition, a prevention strategy for CBI and leukoaraiosis has not yet been established (Leung et al., 2017).

Investigators have increasingly focused on studying the effects of cardiovascular physiology on brain infarctions (Lee et al., 2018; Leung et al., 2017; Yaghi et al., 2018). A recent study has reported that electrocardiogram (ECG) abnormalities are associated with cerebrovascular diseases (Danese et al., 2019; Sawano et al., 2020), while the relationship between ECG parameters and silent vascular brain injury has not been assessed. Understanding the relationship between ECG and silent vascular brain injury may contribute to the improvement of subsequent stroke prevention strategies and provide evidence supporting the notion that cardiac structural or functional abnormalities may predispose patients to silent brain vascular injury (Yaghi et al., 2018). Therefore, the present study aimed to investigate the relationship between ECG profile components and silent vascular brain injury, as defined on MRI. We hypothesized that ECG abnormalities prior to the MRI scans would be associated with a greater risk of incident CBI on the follow‐up MRI, presence of severe white matter hyperintensity and brain atrophy.

## 2 MATERIALS AND METHODS

### 2.1 Study population

This study was conducted as part of the Cardiovascular Health Study (CHS), a multisite, population‐based longitudinal study designed to study cardio‐cerebrovascular diseases in adults aged ≥65 years (Boyle et al., 2021). A total of 5888 participants were recruited from communities beginning in 1989 and followed up for more than 20 years. The institutional review boards at the University of Washington and each study site approved the study and written informed consent was obtained from all participants. The study conforms with World Medical Association Declaration of Helsinki. Details of the CHS have been previously described (Kuller et al., 2016; Yaghi et al., 2018). In the present study, participants with incomplete baseline information and covariates, missing ECG values, and those with a history of stroke, and transient ischemic attack were excluded. Exclusion criteria of the primary analysis are as follows: missing infarct data at the 1st or 2nd visit, infarcts detected on MRI at the 1st visit, and stroke occurrence during follow‐up. In the secondary analysis, participants with available white matter data at the 1st visit were included in the white matter worsening analysis, and those with complete ventricle and sulcal atrophy data on MRI were included in the brain atrophy analysis.

### 2.2 ECG examination

In the primary analysis, ECG abnormalities prior to the 1st MRI scan were used. ECGs were coded according to the Minnesota Code (MC) (Auer et al., 2012). Electrocardiographic abnormalities were defined according to previous publications and MC codes (Auer et al., 2012; Bussink et al., 2013; Denes et al., 2007; Silva et al., 2021). Criteria for ECG abnormalities were summarized as follows: ventricular conduction defect [MC = 7–1, 7–2, or 7–4]; major Q‐wave abnormalities[MC = 1–1 through 1–2, except 1–2–8]; minor Q, QS waves with ST‐T abnormalities[MC = 1–3 or 1–2–8] and [4–1 to 4–3 or 5–1 to 5–3]; isolated ST‐T wave abnormalities[MC = 4–1, 4–2, 5–1, 5–2 without left ventricular (LV) hypertrophy or major Q‐wave abnormalities]; LV hypertrophy, [MC = 3–1, 3–3] and [4–1 to 4–3 or 5–1 to 5–3]; and first‐degree atrioventricular block[MC = 6–3]. Major ECG abnormalities were defined as any of the following: ventricular conduction defect, major Q‐wave abnormalities, LV hypertrophy, isolated ST‐T wave abnormalities, and first‐degree atrioventricular block. Minor ECG abnormalities included the following: minor Q, QS waves [MC = 1−3]; minor Q, QS waves with ST‐T abnormalities [MC = 1–3 or 1–2–8] and [4–1 to 4–3 or 5–1 to 5–3]; high R waves [MC = 3−1, 3−2, 3−3 or 3−4]; minor isolated ST‐T abnormalities [MC = 4−3, 4−4, 5−3 or5−4]; ST elevation [MC = 9−2]; incomplete right bundle branch block [MC = 7–2–1]; long QT interval [QT index ≥110, where QT Index = QT interval × (heart rate+100)/656], short PR [MC = 6−5], right axis deviation [MC = 2−2], and left axis deviation [MC = 2−1].

### 2.3 MRI evaluation

The first brain MRI scans for CHS participants were conducted in years 4–6 (1991−1994) and the second MRI scans were conducted in years 10–11 (1997−1999). Two independent neuroradiologists reviewed the scans using a standard rule to identify the number, size, and location of brain infarcts, as well as the white matter grade scored from 0 (no changes) to 9 (most pronounced changes) (Kalani et al., 2020; Yaghi et al., 2018). Brain atrophy was also measured, considering the sizes of the ventricles and sulci ranging from 0 (smallest) to 9 (largest), as detailed previously (Longstreth et al., 2002; Luoto et al., 2000).

The primary outcome was presence of incident CBIs on the 2nd MRI examination, excluding those with an infarct detected on the 1st MRI and stroke occurrence before the 2nd MRI exam. CBI on MRI was defined as an area of abnormal signal ≥3 mm in diameter within one vascular distribution with no mass effect. Those with positive MRI findings must be asymptomatic. Secondary outcomes included white matter, ventricular, and sulcal atrophy on the 1st MRI. Severe white matter hyperintensity was defined as grades 3−9. Severe brain atrophy was defined as grades 3−9 atrophy of the ventricle or sulci (Rosano et al., 2005).

### 2.4 Covariates

The latest information on the covariates for the primary and secondary analyses was collected before the initial MRI. These variables included age, sex, race, body mass index, systolic blood pressure, antihypertensive drug therapy, smoking status, diabetes mellitus, congestive heart failure, myocardial infarction, and atrial fibrillation, as previously defined (Longstreth et al., 2002; Longstreth et al., 2005).

### 2.5 Statistical analysis

Continuous variables are presented as means (standard deviations), and categorical variables are reported as frequencies (percentages). Differences in baseline characteristics between the groups were tested using the Student's *t*‐test for continuous variables and the chi‐square test for categorical variables.

In the primary analysis, a logistic regression model with a prospective cohort study design was used to assess the relationship between all measured ECG abnormalities 4 years before the 1st MRI scan and presence of incident CBIs on the 2nd MRI scan.

Secondary analyses were conducted in a cross‐sectional design. We further evaluated the relationship between measured ECG abnormalities at baseline, severe white matter hyperintensity, and severe brain atrophy on the 1st MRI scan with logistic regression models. A multiple linear regression model was used to assess the association among ECG abnormalities, degree of white matter hyperintensity and brain atrophy grade. Sensitivity analyses were conducted to examine the association of ECG abnormalities before 1st MRI scan with presence of incident CBI on the 2nd MRI scan, excluding participants who had hypertension and atrial fibrillation participants. Because numerous studies provided evidence that hypertension was associated with CBI as well as ischemic stroke and hypertension was the most important risk factor for covert vascular brain injury (Cannistraro et al., 2019; Kalani et al., 2020; Leung et al., 2017; Wardlaw et al., 2013). Atrial fibrillation is the most common cardiac factor related with stroke occurrence. Previous studies ever thought that atrial fibrillation may influence the association between atrial disease and CBI (Kamel et al., 2015; Yaghi et al., 2018). A prior study ever performed sensitivity analysis that excluded participants with atrial fibrillation when evaluating the relationship between left atrial abnormality and vascular brain injury (Kamel et al., 2015).

Furthermore, exploratory analysis was performed to evaluate the association between ECG abnormalities before 1st MRI scan and clinically defined nonlacunar ischemic stroke occurrence before the 2nd MRI scan with a logistic regression model. Only the first occurrence of nonlacunar ischemic stroke was included. The first‐time nonlacunar ischemic stroke occurred after 1st MRI scan and before the 2nd MRI scan. Adjustment covariates were specified a priori and included age, sex, race, body mass index, systolic blood pressure, antihypertensive drug therapy, smoking status, diabetes mellitus, congestive heart failure, myocardial infarction, atrial fibrillation, left atrial dimension, and all ECG‐measured parameters in all analyses. Statistical significance was set at *p* < .05. Statistical analyses were conducted using the SPSS ver. 24.0 and R ver. 3.5.3.

## 3 RESULTS

**FIGURE 1:**
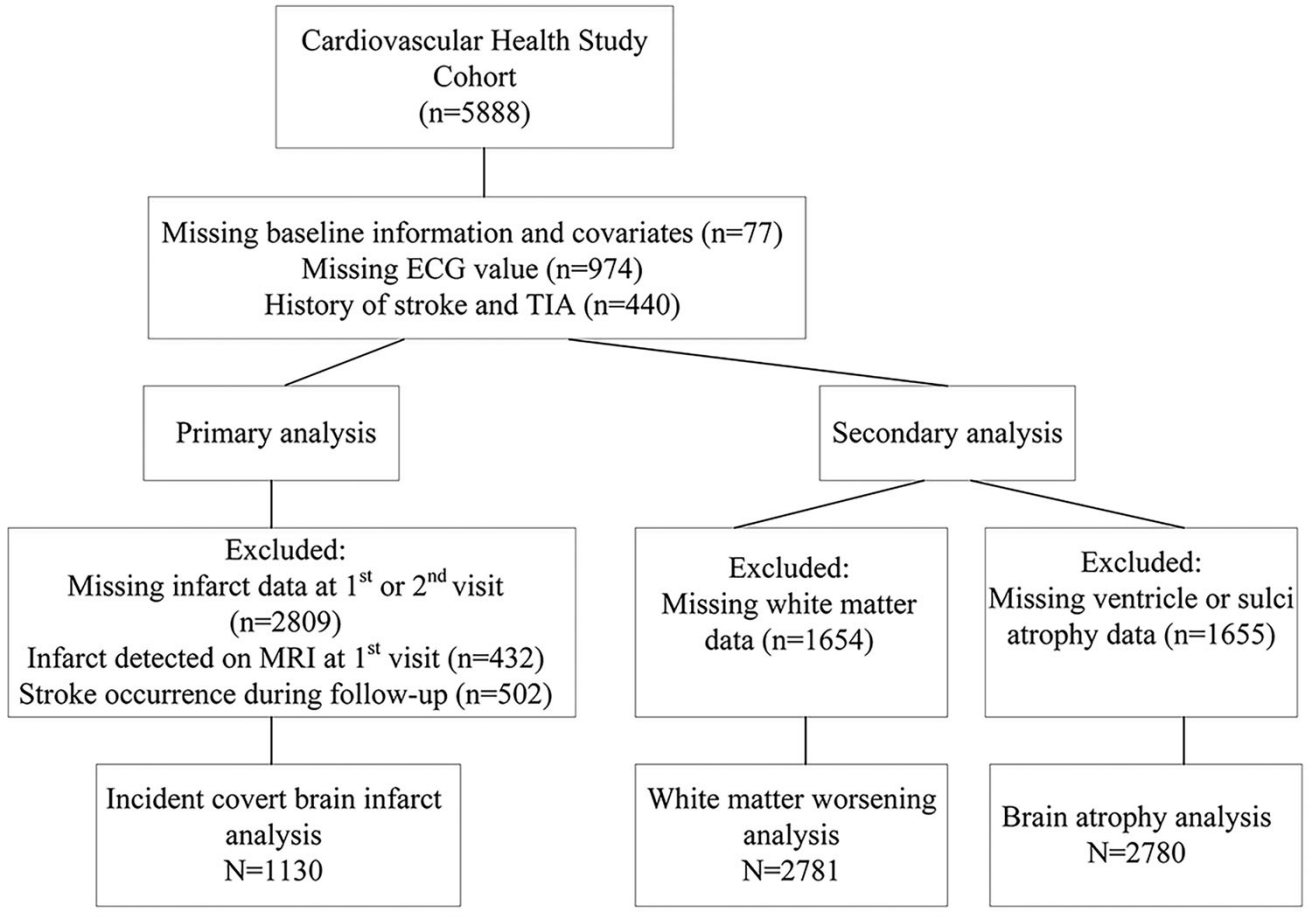
The selection process of the study population.

A total of 5888 participants were enrolled in the CHS study. The selection process of the study population is shown in Figure 1. A total of 1130 participants were eligible for the primary analysis, 2781 for the white matter analysis, and 2780 for the brain atrophy analysis. The baseline information of the participants included in the primary and secondary analyses is presented in Table 1. In the primary analysis, no significant differences were detected between the two groups. The mean age was 69.7 ± 4.1 years and 40.4% were men. A total of 207 participants (18.3%) had covert infarcts. A total of 965 individuals (34.7%) had severe white matter hyperintensity and 2478 individuals (89.1%) had severe brain atrophy.

**TABLE 1:** Basic characteristics of all participants in our study cohort.

		Primary analysis			Secondary analysis					
	Total	Incident covert brain infarct	No incident covert brain infarct	*p* value	White matter grade 3–9	White matter grade 0–2	*p* value	Brain atrophy grade 3–9	Brain atrophy grade 0–2	*p* Value
Variables	1130	207	923		965	1816		2478	302	
Age	69.7 (4.1)	70.2 (4.3)	69.6 (4.1)	.050	72.6 (5.3)	70.3 (4.6)	<.001	71.4 (5)	68.4 (3.5)	<.001
Male	457 (40.4%)	83 (40.1%)	374 (40.5%)	.911	375 (38.9%)	790 (43.5%)	.018	1100 (44.4%)	66 (21.9%)	<.001
Race										.151
White	1089 (96.4%)	202 (97.6%)	887 (96.1%)	.414	915 (94.8%)	1745 (96.1%)	.184	2364 (95.4%)	295 (97.7%)	
Black	35 (3.1%)	5 (2.4%)	30 (3.3%)		44 (4.6%)	66 (3.6%)		103 (4.2%)	7 (2.3%)	
Other	6 (0.53)	0 (0%)	6 (0.7%)		6 (0.6%)	5 (0.3%)		11 (0.4%)	0 (0%)	
High school or higher education	895 (79.3%)	160 (77.3%)	735 (79.8%)	.420	696 (72.3%)	1404 (77.5%)	.003	1878 (76%)	220 (73.1%)	.271
**Vascular risk factors**										
Smoking				.746			.897			<.001
Never smoker	568 (50.3%)	101 (48.8%)	467 (50.6%)		465 (48.2%)	862 (47.5%)		1153 (46.5%)	173 (57.3%)	
Former smoker	455 (40.3%)	88 (42.5%)	367 (39.8%)		399 (41.3%)	755 (41.6%)		1059 (42.7%)	95 (31.5%)	
Current smoker	107 (9.4%)	18 (8.7%)	89 (9.6%)		101 (10.5%)	199 (11%)		266 (10.7%)	34 (11.3%)	
Alcohol drinking	724 (64.5%)	124 (60.5%)	600 (65.4%)	.188	561 (58.4%)	1145 (63.4%)	.010	1538 (62.3%)	166 (55.3%)	.018
Physical activity				.260			.001			.128
No exercise	50 (4.4%)	9 (4.3%)	41 (4.4%)		77 (8%)	100 (5.5%)		163 (6.6%)	14 (4.6%)	
Low exercise	467 (41.4%)	98 (47.3%)	369 (40%)		462 (47.9%)	791 (43.6%)		1100 (44.4%)	152 (50.3%)	
Moderate exercise	440 (39.0%)	70 (33.8%)	370 (40.1%)		331 (34.3%)	679 (37.4%)		902 (36.4%)	107 (35.4%)	
High exercise	172 (15.2%)	30 (14.5%)	142 (15.4%)		95 (9.8%)	244 (13.5%)		311 (12.6%)	29 (9.6%)	
BMI	26.2 (3.7)	25.9 (3.4)	26.2 (3.7)	.352	25.9 (3.6)	26.2 (3.8)	.098	26.1 (3.7)	26.4 (4)	.209
LDL	133 (34.6)	130.5 (34.8)	133.5 (34.5)	.299	133.3 (35.5)	133.2 (34.7)	.921	132.6 (34.8)	138.2 (36.3)	.014
HDL	55.6 (16.6)	55.6 (17)	55.5 (16.5)	.895	55 (15.1)	54.6 (16.2)	.088	54.5 (15.9)	56 (14.7)	.035
**Medical history**										
Hypertension	568 (34.0%)	110 (53.1%)	458 (49.6%)	.360	642 (66.5%)	1002 (55.2%)	<.001	1478 (59.6%)	162 (53.6%)	.045
Diabetes mellitus	106 (9.4%)	20 (9.7%)	86 (9.3%)	.878	129 (13.4%)	201 (11.1%)	.074	301 (12.1%)	30 (9.9%)	.262
Atrial fibrillation	36 (3.2%)	9 (4.3%)	27 (2.9%)	.292	43 (4.5%)	80 (4.4%)	.951	109 (4.4%)	13 (4.3%)	.940
Heart failure	13 (1.2%)	3 (1.4%)	10 (1.1%)	.656	23 (2.4%)	34 (1.9%)	.365	54 (2.2%)	2 (0.7%)	.076
Myocardial infarction	55 (4.9%)	11 (5.3%)	44 (4.8%)	.741	90 (9.3%)	125 (6.9%)	.022	197 (7.9%)	15 (5%)	.065
**Medication**										
Anticoagulants use	4 (0.4%)	1 (0.5%)	3 (0.3%)	.729	3 (0.3%)	11 (0.6%)	.296	14 (0.6%)	0 (0%)	.190
Aspirin use	24 (2.1%)	3 (1.4%)	21 (2.3%)	.456	19 (2%)	49 (2.7%)	.236	63 (2.5%)	5 (1.7%)	.346
Antihypertensive medications	384 (40%)	65 (31.4%)	319 (34.6%)	.386	454 (47%)	672 (37%)	<.001	1019 (41.1%)	104 (34.4%)	.025

BMI, body mass index; LDL, low‐density lipoprotein; HDL, high‐density lipoprotein.

**FIGURE 2:**
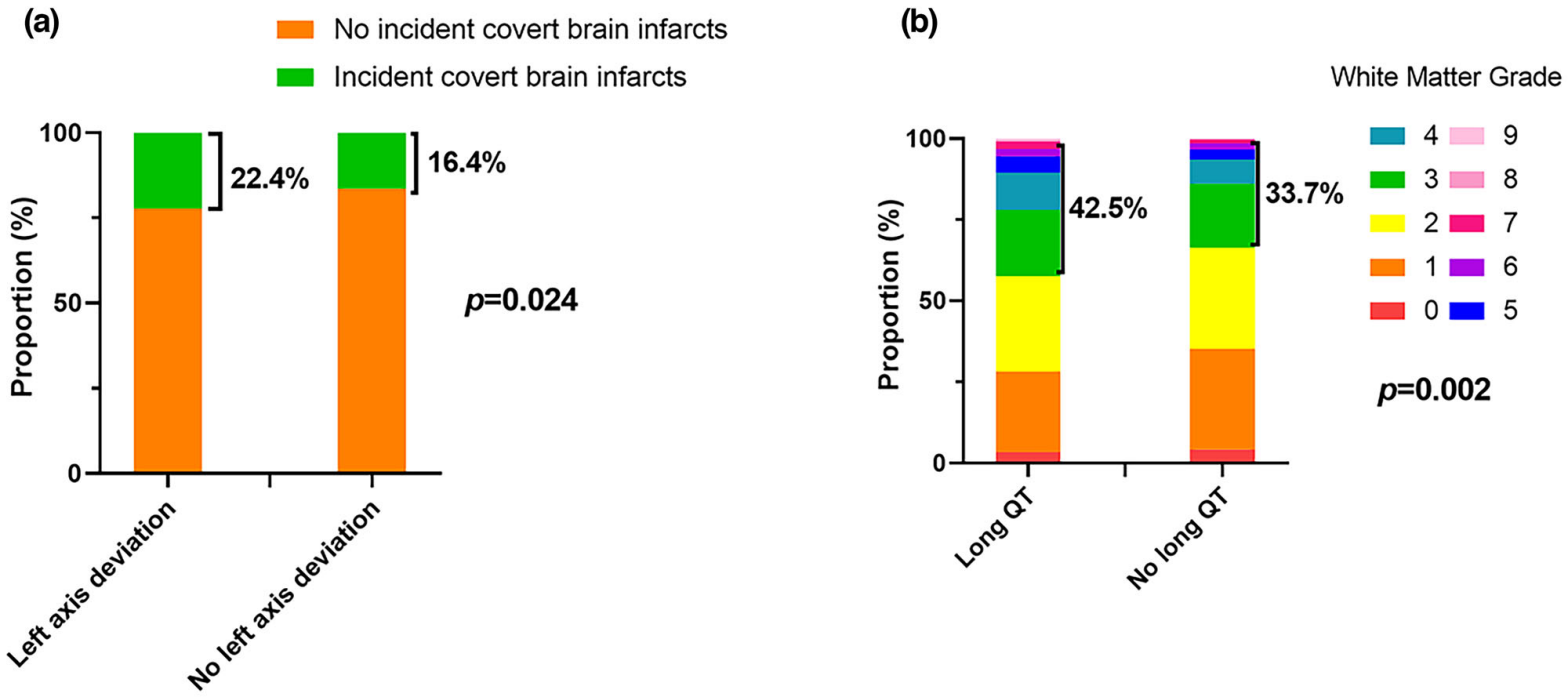
Different proportions of ECG abnormality in different outcomes. (a) The proportion of left axis deviation in populations with and without incident covert brain infarct. (b) The proportion of long QT interval in different white matter grades. Chi‐square tests were used.

The frequency distribution of ECG abnormalities in the incidence of CBI and different white matter grades is shown in Figure 2. The percentage of covert infarcts in the left axis deviation group was higher than that in the no left axis deviation population. Participants with long QT intervals on ECG accounted for a higher percentage of white matter grades 3−9 than those with normal QT intervals.

The main results are listed in Table 2. Left axis deviation measured at year 4 was associated with presence of incident CBI on the 2nd MRI scan from a fully adjusted model in Table 2. Other ECG indicators were not significantly related to the risk of covert brain infarcts. Sensitivity analyses were performed excluding hypertensive and atrial fibrillation participants (Supplemental Table S1). Data show a strong relationship between left axis deviation and risk of covert brain infarcts after full adjustment (OR, 1.79, 95% CI: 1.04−3.10, *p* = .037). Minor isolated ST‐T abnormalities were related with incident stroke in a fully adjusted model (OR, 1.51, 95% CI: 1.03–2.21, *p* = .036, Supplemental Table S2).

**TABLE 2:** Relationship between ECG abnormalities and covert brain infarcts.

Primary outcome	Incident covert brain infarcts	
	*n* = 207/1130	
	OR (95% CI)	*p*
**Major ECG abnormalities before 1st MRI scan**		
First‐degree atrioventricular block	1.36 (0.70–2.64)	.362
Left ventricular hypertrophy	2.92 (0.92–9.29)	.069
Ventricular conduction defect	1.45 (0.71–2.94)	.308
Major Q‐wave abnormalities	1.34 (0.61–2.91)	.464
Isolated ST‐T wave abnormalities	0.83 (0.34–2.06)	.689
**Minor ECG abnormalities before 1st MRI scan**		
Minor Q, QS waves	1.09 (0.51–2.36)	.821
High R waves	1.11 (0.50–2.47)	.805
Minor isolated ST‐T abnormalities	0.71 (0.38–1.32)	.282
ST elevation†	–	.995
Incomplete RBBB	0.90 (0.36–2.26)	.827
Long QT interval	0.60 (0.30–1.18)	.142
Short PR	2.17 (0.54–8.78)	.276
Left axis deviation	1.45 (1.01–2.08)	.047
Right axis deviation	0.43 (0.12–1.56)	.200
Minor Q, QS waves with ST‐T abnormalities	1.19 (0.30–4.79)	.808

†The number of participants with ST elevation was 6, so the confidence interval was too wide to show it. Models adjusted for age, sex, race, body mass index, systolic blood pressure, antihypertensive drug therapy, smoking status, diabetes mellitus, congestive heart failure, myocardial infarction, atrial fibrillation, left atrial dimension, and all ECG‐measured parameters.

A long QT interval was associated with severe white matter hyperintensity after full adjustment (Table 3). The relationship between ECG abnormalities and white matter and brain atrophy grades is listed in Table 4. A long QT interval was positively associated with the white matter grade after full adjustment. Minor Q and QS waves with ST‐T abnormalities were positively related to sulcal atrophy after full adjustment. None of the parameters were found to be associated with ventricular atrophy.

**TABLE 3:** Relationship between ECG abnormalities with severe white matter hyperintensity and brain atrophy.

Secondary outcomes	White matter grade 3–9		Brain atrophy 3–9	
	965/2781		*n* = 2478/2780	
	OR (95% CI)	*p*	OR (95% CI)	*p*
**Major ECG abnormalities**				
First‐degree atrioventricular block	0.93 (0.63–1.38)	.719	0.76 (0.41–1.40)	.380
Left ventricular hypertrophy	1.54 (0.94–2.52)	.088	1.72 (0.58–5.07)	.327
Ventricular conduction defect	0.73 (0.50–1.07)	.103	1.37 (0.71–2.65)	.353
Major Q‐wave abnormalities	1.35 (0.87–2.09)	.178	1.43 (0.61–3.34)	.409
Isolated ST‐T wave abnormalities	0.86 (0.57–1.30)	.471	0.88 (0.47–1.66)	.695
Minor ECG abnormalities				
Minor Q, QS waves	1.21 (0.76–1.91)	.424	2.40 (0.85–6.79)	.098
High R waves	0.95 (0.62–1.45)	.805	0.87 (0.48–1.61)	.672
Minor isolated ST‐T abnormalities	1.20 (0.92–1.57)	.171	1.24 (0.81–1.92)	.328
ST elevation	1.41 (0.53–3.77)	.489	0.70 (0.15–3.25)	.650
Incomplete RBBB	0.94 (0.52–1.69)	.837	0.57 (0.28–1.16)	.121
Long QT interval	1.36 (1.04–1.77)	.024	0.90 (0.59–1.36)	.600
Short PR	0.98 (0.49–1.99)	.960	1.11 (0.42–2.97)	.835
Left axis deviation	0.97 (0.79–1.18)	.736	0.82 (0.61–1.11)	.202
Right axis deviation	0.68 (0.40–1.16)	.156	0.74 (0.36–1.51)	.406
Minor Q, QS waves with ST‐T abnormalities	1.16 (0.57–2.35)	.684	4.23 (0.56–32.16)	.163

Models adjusted for age, sex, race, body mass index, systolic blood pressure, antihypertensive drug therapy, smoking status, diabetes mellitus, congestive heart failure, myocardial infarction, atrial fibrillation, left atrial dimension, and all ECG‐measured parameters.

**TABLE 4:** Relationship between ECG abnormalities with continuous white matter grade, ventricle atrophy grade and sulci atrophy grade.

Secondary outcomes	White matter grade		Ventricle atrophy		Sulci atrophy	
	β (95% CI)	*p*	β (95% CI)	*p*	β (95% CI)	*p*
**Major ECG abnormalities**						
First‐degree atrioventricular block	–0.05 (−0.28 to 0.18)	.661	−0.02 (−0.24 to 0.18)	.810	0.02 (−0.17 to 0.22)	.821
Left ventricular hypertrophy	0.18 (−0.12 to 0.49)	.236	0.01 (−0.26 to 0.29)	.923	0.03 (−0.23 to 0.29)	.816
Ventricular conduction defect	−0.07 (−0.29 to 0.14)	.507	−0.07 (−0.27 to 0.12)	.467	0.09 (−0.09 to 0.28)	.341
Major Q‐wave abnormalities	0.16 (−0.11 to 0.43)	.245	0.10 (−0.14 to 0.35)	.404	−0.01 (−0.24 to 0.21)	.928
Isolated ST‐T wave abnormalities	0.02 (−0.22 to 0.27)	.855	0.04 (−0.18 to 0.26)	.731	0.06 (−0.14 to 0.27)	.532
**Minor ECG abnormalities**						
Minor Q, QS waves	0.08 (−0.19 to 0.36)	.547	0.16 (−0.09 to 0.42)	.204	−0.05 (−0.29 to 0.18)	.637
High R waves	0.08 (−0.16 to 0.33)	.514	−0.09 (−0.32 to 0.14)	.441	−0.18 (−0.39 to 0.03)	.097
Minor isolated ST‐T abnormalities	0.13 (−0.02 to 0.30)	.103	0.01 (−0.13 to 0.16)	.835	−0.01 (−0.15 to 0.12)	.821
ST elevation	0.15 (−0.44 to 0.76)	.612	−0.38 (−0.93 to 0.16)	.173	−0.15 (−0.66 to 0.35)	.546
Incomplete RBBB	0.04 (−0.29 to 0.39)	.781	−0.19 (−0.50 to 0.11)	.219	−0.24 (−0.52 to 0.05)	.101
Long QT interval	0.23 (0.07 to 0.40)	.004	0.13 (−0.01 to 0.28)	.074	−0.06 (−0.20 to 0.07)	.343
Short PR	0.03 (−0.37 to 0.45)	.863	0.18 (−0.19 to 0.55)	.346	0.09 (−0.25 to 0.44)	.591
Left axis deviation	−0.02 (−0.14 to 0.09)	.652	−0.02 (−0.13 to 0.08)	.648	0.01 (−0.08 to 0.11)	.821
Right axis deviation	−0.23 (−0.52 to 0.05)	.108	−0.006 (−0.26 to 0.25)	.962	−0.09 (−0.33 to 0.15)	.463
Minor Q, QS waves with ST‐T abnormalities	0.11 (−0.32 to 0.55)	.612	0.38 (−0.02 to 0.78)	.063	0.43 (0.06 to 0.81)	.023

Models adjusted for age, sex, race, body mass index, systolic blood pressure, antihypertensive drug therapy, smoking status, diabetes mellitus, congestive heart failure, myocardial infarction, atrial fibrillation, left atrial dimension, and all ECG‐measured parameters.

## 4 DISCUSSION

In this community‐dwelling population‐based longitudinal study, left axis deviation at baseline was associated with following presence of incident CBIs in a stroke‐free elderly population. A long QT interval at baseline was associated with severe white matter hyperintensity. A positive relationship was observed between minor Q and QS waves with ST‐T abnormalities at baseline and sulcal atrophy grade.

The relationship between cardiac physiological indicators and covert cerebrovascular abnormalities has been widely reported (Cannistraro et al., 2019; Godin et al., 2011; Gottesman et al., 2010; Kamel et al., 2015; Lee et al., 2018; Leung et al., 2017; Wardlaw et al., 2013; Yaghi et al., 2018). In particular, blood pressure has been demonstrated to be related to covert brain infarction and white matter hyperintensities on MRI (Godin et al., 2011; Gottesman et al., 2010; Kamel et al., 2015; Leung et al., 2017; Wardlaw et al., 2013). The mean right atrial pressure was likewise associated with white matter hyperintensities (Lee et al., 2018). Another study found that a larger left atrial diameter was related to brain infarcts (Yaghi et al., 2018). Regarding ECG examination, one study reported that isolated nonspecific ST segment and T wave abnormalities were related to a higher risk of ischemic stroke (Sawano et al., 2020). Another cohort study found that a silent myocardial infarction detected on ECG was associated with incident ischemic stroke (Merkler et al., 2021). However, evidence regarding the link between ECG parameters and subclinical vascular brain injury on MRI is lacking.

The primary finding of the present study was that left axis deviation may be related with high risk of covert brain infarcts in the stroke‐free elderly population. The relationship between left axis deviation and presence of incident CBIs could be due to several factors. First, the total activation time may be longer in the population with a left axis deviation. Thus, activation of the basal anterolateral region may be delayed (Abu‐Alrub et al., 2021). Second, left axis deviation was related to left‐sided structural abnormalities, specifically LV diastolic and systolic dysfunction, as well as LV dilation (Lui et al., 2021). These two reasons may cause a decrease in the flow velocity in the left ventricle and cerebral blood flow, thereby contributing to stasis and ischemia of small‐vessel territories and brain infarcts (Cannistraro et al., 2019; Di Tullio et al., 1999; Nakanishi et al., 2017; Wardlaw et al., 2013; Yaghi et al., 2018). This result was consistent with a previous study that reported that LV concentric hypertrophy carries a higher independent risk for silent brain infarcts in a multiethnic stroke‐free general population (Nakanishi et al., 2017). Another potential explanation may be that left axis deviation and CBI share vascular risk factors, which are the causes of brain infarcts (Yaghi et al., 2018). After excluding participants with hypertension, the association between left axis deviation and covert brain infarcts was more apparent. It may be inferred that the pathology of silent vascular brain injury caused by left axis deviation is different from that of hypertension (Cannistraro et al., 2019). There may be several uncertain pathological conditions that can lead to a silent vascular brain injury. Although two other studies reported that a left atrial abnormality was related to prevalent brain infarcts, our results provide further evidence that a cardiac structural or functional abnormality may occur before silent brain infarcts. Moreover, when we analyzed the association between ECG abnormalities and following ischemic stroke occurrence, left axis deviation was not related with risk of stroke. It may indicate that the risk factors of stroke and silent vascular brain injury may be different in the same time period, which may be related to the disease progression.

A long QT interval was found to be associated with severe white matter hyperintensity. A recent study included patients from the outpatient Cognitive Impairment and Dementia Center of a hospital and reported that a prolonged QT interval was prevalent in patients with dementia and in patients with higher leukoaraiosis scale scores (Danese et al., 2019). Although the community population in our study was different from that of the other study, our results confirmed the results of the latter with larger sample size. A previous study suggested that cerebral vascular load is associated with prolonged QTc (Danese et al., 2019). Patients with lesions in autonomic cardiac centers might have a change in the vascular origin, which causes unstable blood pressure with subsequent dysfunction of cerebral perfusion and alteration of the white matter (Dias et al., 2013). Previous studies found that a silent myocardial infarction was associated with the risk of stroke (Merkler et al., 2021; Merkler et al., 2019). However, the relationship between silent myocardial infarction and silent vascular brain injury is unknown. Our study found that minor Q and QS waves with ST‐T abnormalities were positively related to the sulcal atrophy grade. Minor Q and QS waves with ST‐T abnormalities belong to silent myocardial infarction (Gibson et al., 2018). One possible explanation may be that silent myocardial infarction indicates ischemic changes in vessels secondary to endothelial dysfunction and impaired autoregulation, which may also lead to small‐vessel diseases, including sulcal atrophy, detected on MRI (Cannistraro et al., 2019; Wardlaw et al., 2013).

However, some limitations still need to be considered. First, each participant underwent MRI twice during the entire follow‐up period. We could not acquire dynamic changes in brain vascular findings on MRI. Participants who underwent both MRI scans may be healthier than those who did not, limiting generalizability. Second, the populations in the different analyses were not consistent. There were different missing values regarding covert brain infarcts, white matter, and brain atrophy on MRI. The results may have been altered if all participants had completed both MRI scans and were included in both analyses (Longstreth et al., 2002; Longstreth et al., 2005). Third, routine ECG examination was not performed in all participants in the CHS. Fourth, our study was performed in an elderly population aged ≥65 years, and the results may differ in younger populations (Merkler et al., 2021). Last, there may be uncontrolled confounding factors in the study, such as undiagnosed atrial fibrillation and imaging interrater variability over the time scale.

## 5 CONCLUSION

In a stroke‐free community‐dwelling population, our study found that left axis deviation may occur before covert brain infarcts. We likewise found that a long QT interval was associated with severe white matter hyperintensity and that minor Q and QS waves with ST‐T abnormalities were positively related to sulcal atrophy. These results may reveal novel risk factors and explain the causes of silent vascular brain injuries. Further studies are needed to determine the detailed pathology of small‐vessel disease lesions on MRI.

## AUTHOR CONTRIBUTIONS

FG and JH conceived and designed the research; FG, CC, and FL organized the data; FG and CC performed the data analysis; all authors interpreted the results of statistical analysis; FG, CC, and FL wrote the manuscript; JH revised the manuscript critically. All the authors have reviewed the manuscript.

## CONFLICT OF INTEREST STATEMENT

The authors have no relevant financial or nonfinancial interests to disclose.

### ETHICS STATEMENT

The institutional review boards at the University of Washington and each study site approved the study and written informed consent was obtained from all participants.

### PEER REVIEW

The peer review history for this article is available at https://publons.com/publon/10.1002/brb3.2991.

## Supporting information

Supplementary material

Supplementary material

## Data Availability

The data that support the findings of this study are available in Biologic Specimen and Data Repositories Information Coordinating Center (BioLINCC) at https://biolincc.nhlbi.nih.gov/studies/chs/.

## References

[brb32991-bib-0001] Abu‐Alrub, S. , Strik, M. , Huntjens, P. , Ramirez, F. D. , Potse, M. , Cochet, H. , Marchand, H. , Buliard, S. , Eschalier, R. , Haã¯Ssaguerre, M. , Bordachar, P. , & Ploux, S. (2021). Left‐axis deviation in patients with nonischemic heart failure and left bundle branch block is a purely electrical phenomenon. Heart Rhythm, 18, 1352–1360. https://doi.10.1016/j.hrthm.2021.03.042 33831543 10.1016/j.hrthm.2021.03.042

[brb32991-bib-0002] Appleton, J. P. , Woodhouse, L. J. , Adami, A. , Becker, J. L. , Berge, E. , Cala, L. A. , Casado, A. M. , Caso, V. , Christensen, H. K. , Dineen, R. A. , Gommans, J. , Koumellis, P. , Szatmari, S. , Sprigg, N. , Bath, P. M. , & Wardlaw, J. M. (2020). Imaging markers of small vessel disease and brain frailty, and outcomes in acute stroke. Neurology, 94, e439–e452. https://doi.10.1212/WNL.0000000000008881 31882527 10.1212/WNL.0000000000008881 PMC7080284

[brb32991-bib-0003] Auer, R. , Bauer, D. C. , Marques‐Vidal, P. , Butler, J. , Min, L. J. , Cornuz, J. , Satterfield, S. , Newman, A. B. , Vittinghoff, E. , & Rodondi, N. , Health ABC Study . (2012). Association of major and minor ECG abnormalities with coronary heart disease events. JAMA, 307, 1497–1505. https://doi.10.1001/jama.2012.434 22496264 10.1001/jama.2012.434 PMC4006989

[brb32991-bib-0004] Boyle, C. P. , Raji, C. A. , Erickson, K. I. , Lopez, O. L. , Becker, J. T. , Gach, H. M. , Kuller, L. H. , Longstreth, W. , Carmichael, O. T. , Riedel, B. C. , & Thompson, P. M. (2021). Estrogen, brain structure, and cognition in postmenopausal women. Human Brain Mapping, 42, 24–35. https://doi.10.1002/hbm.25200 32910516 10.1002/hbm.25200 PMC7721237

[brb32991-bib-0005] Bussink, B. E. , Holst, A. G. , Jespersen, L. , Deckers, J. W. , Jensen, G. B. , & Prescott, E. (2013). Right bundle branch block: Prevalence, risk factors, and outcome in the general population: Results from the Copenhagen city heart study. European Heart Journal, 34, 138–146. https://doi.10.1093/eurheartj/ehs291 22947613 10.1093/eurheartj/ehs291

[brb32991-bib-0006] Cannistraro, R. J. , Badi, M. , Eidelman, B. H. , Dickson, D. W. , Middlebrooks, E. H. , & Meschia, J. F. (2019). CNS small vessel disease: A clinical review. Neurology, 92, 1146–1156. https://doi.10.1212/WNL.0000000000007654 31142635 10.1212/WNL.0000000000007654 PMC6598791

[brb32991-bib-0007] Danese, A. , Federico, A. , Martini, A. , Mantovani, E. , Zucchella, C. , Tagliapietra, M. , Tamburin, S. , Cavallaro, T. , Marafioti, V. , Monaco, S. , & Turri, G. (2019). QTc prolongation in patients with dementia and mild cognitive impairment: Neuropsychological and brain imaging correlations. Journal of Alzheimer's Disease, 72, 1241–1249. https://doi.10.3233/JAD‐190632 10.3233/JAD-190632 31683480

[brb32991-bib-0008] Denes, P. , Larson, J. C. , Lloyd‐Jones, D. M. , Prineas, R. J. , & Greenland, P. (2007). Major and minor ECG abnormalities in asymptomatic women and risk of cardiovascular events and mortality. Journal of the American Medical Association, 297, 978–985. 10.1001/jama.297.9.978 17341712

[brb32991-bib-0009] Dias, F. L. D. a. C. , Silva, R. M. F. L. D. a. , Moraes, E. N. D. e. , & Caramelli, P. (2013). Clinical and autonomic profile of patients with Alzheimer's disease and mixed dementia patients. Revista Da Associacao Medica Brasileira, 59, 435–441. https://doi.10.1016/j.ramb.2013.04.004 24119378 10.1016/j.ramb.2013.04.004

[brb32991-bib-0010] Di Tullio, M. R. , Sacco, R. L. , Sciacca, R. R. , & Homma, S. (1999). Left atrial size and the risk of ischemic stroke in an ethnically mixed population. Stroke; A Journal of Cerebral Circulation, 30, 2019–2024. https://doi.10.1161/01.Str.30.10.2019 10.1161/01.str.30.10.2019 10512901

[brb32991-bib-0011] Gibson, C. M. , Nafee, T. , & Kerneis, M. (2018). Silent myocardial infarction: Listen to the evidence. Journal of the American College of Cardiology, 71, 9–11. https://doi.10.1016/j.jacc.2017.10.069 29301632 10.1016/j.jacc.2017.10.069

[brb32991-bib-0012] Godin, O. ©. L. , Tzourio, C. , Maillard, P. , Mazoyer, B. , & Dufouil, C. (2011). Antihypertensive treatment and change in blood pressure are associated with the progression of white matter lesion volumes the three‐city (3C)‐Dijon magnetic resonance imaging study. Circulation, 123, 266–273. https://doi.10.1161/Circulationaha.110.961052 21220733 10.1161/CIRCULATIONAHA.110.961052

[brb32991-bib-0013] Gottesman, R. F. , Coresh, J. , Catellier, D. J. , Sharrett, A. R. , Rose, K. M. , Coker, L. H. , Shibata, D. K. , Knopman, D. S. , Jack, C. R. , & Mosley, T. H. (2010). Blood pressure and white‐matter disease progression in a biethnic cohort atherosclerosis risk in communities (ARIC) study. Stroke; A Journal of Cerebral Circulation, 41, 3–8. https://doi.10.1161/Strokeaha.109.566992 10.1161/STROKEAHA.109.566992 PMC2803313 19926835

[brb32991-bib-0014] Kalani, R. , Bartz, T. M. , Suchy‐Dicey, A. , Elkind, M. S. V. , Psaty, B. M. , Leung, L. Y. , Rice, K. , Tirschwell, D. , & Longstreth, W. T. (2020). Cholesterol variability and cranial magnetic resonance imaging findings in older adults: The cardiovascular health study. Stroke; A Journal of Cerebral Circulation, 51, 69–74. https://doi.10.1161/STROKEAHA.119.026698 10.1161/STROKEAHA.119.026698 PMC7000173 31842691

[brb32991-bib-0015] Kamel, H. , Bartz, T. M. , Longstreth, W. T. , Okin, P. M. , Thacker, E. L. , Patton, K. K. , Stein, P. K. , Gottesman, R. F. , Heckbert, S. R. , Kronmal, R. A. , Elkind, M. S. , & Soliman, E. Z. (2015). Association between left atrial abnormality on ECG and vascular brain injury on MRI in the cardiovascular health study. Stroke; A Journal of Cerebral Circulation, 46, 711–716. 10.1161/STROKEAHA.114.007762 PMC4342300 25677594

[brb32991-bib-0016] Kuller, L. H. , Lopez, O. L. , Mackey, R. H. , Rosano, C. , Edmundowicz, D. , Becker, J. T. , & Newman, A. B. (2016). Subclinical cardiovascular disease and death, dementia, and coronary heart disease in patients 80+ years. Journal of the American College of Cardiology, 67, 1013–1022. https://doi.10.1016/j.jacc.2015.12.034 26940919 10.1016/j.jacc.2015.12.034 PMC5502352

[brb32991-bib-0017] Lee, W. ‐J. , Jung, K. ‐H. , Ryu, Y. J. , Kim, J. ‐M. , Lee, S. ‐T. , Chu, K. , Kim, M. , Lee, S. K. , & Roh, J. ‐K. (2018). Association of cardiac hemodynamic factors with severity of white matter hyperintensities in chronic valvular heart disease. JAMA Neurology, 75, 80–87. https://doi.10.1001/jamaneurol.2017.2853 29114731 10.1001/jamaneurol.2017.2853 PMC5833485

[brb32991-bib-0018] Leung, L. Y. , Bartz, T. M. , Rice, K. , Floyd, J. , Psaty, B. , Gutierrez, J. , Longstreth, W. T. , & Mukamal, K. J. (2017). Blood pressure and heart rate measures associated with increased risk of covert brain infarction and worsening leukoaraiosis in older adults. Arteriosclerosis, Thrombosis, and Vascular Biology, 37, 1579–1586. https://doi.10.1161/ATVBAHA.117.309298 28663254 10.1161/ATVBAHA.117.309298 PMC5551454

[brb32991-bib-0019] Longstreth, W. T. , Arnold, A. M. , Beauchamp, N. J. , Manolio, T. A. , Lefkowitz, D. , Jungreis, C. , Hirsch, C. H. , O'Leary, D. H. , & Furberg, C. D. (2005). Incidence, manifestations, and predictors of worsening white matter on serial cranial magnetic resonance imaging in the elderly—The cardiovascular health study. Stroke; A Journal of Cerebral Circulation, 36, 56–61. 10.1161/01.Str.0000149625.99732.69 15569873

[brb32991-bib-0020] Longstreth, W. T., Jr. , Arnold, A. M. , Beauchamp, N. J., Jr. , Manolio, T. A. , Lefkowitz, D. , Jungreis, C. , Hirsch, C. H. , O'Leary, D. H. , & Furberg, C. D. (2005). Incidence, manifestations, and predictors of worsening white matter on serial cranial magnetic resonance imaging in the elderly: The cardiovascular health study. Stroke; A Journal of Cerebral Circulation, 36, 56–61. 10.1161/01.STR.0000149625.99732.69 15569873

[brb32991-bib-0021] Longstreth, W. T. , Dulberg, C. , Manolio, T. A. , Lewis, M. R. , Beauchamp, N. J. , O'Leary, D. , Carr, J. , & Furberg, C. D. (2002). Incidence, manifestations, and predictors of brain infarcts defined by serial cranial magnetic resonance imaging in the elderly: The cardiovascular health study. Stroke; A Journal of Cerebral Circulation, 33, 2376–2382. https://doi.10.1161/01.str.0000032241.58727.49 10.1161/01.str.0000032241.58727.49 12364724

[brb32991-bib-0022] Longstreth, W. T. , Dulberg, C. , Manolio, T. A. , Lewis, M. R. , Beauchamp, N. J. , O'Leary, D. , Carr, J. , & Furberg, C. D. (2002). Incidence, manifestations, and predictors of brain infarcts defined by serial cranial magnetic resonance imaging in the elderly—The cardiovascular health study. Stroke; A Journal of Cerebral Circulation, 33, 2376–2382. 10.1161/01.Str.0000032241.58727.49 12364724

[brb32991-bib-0023] Lui, J. K. , Sangani, R. A. , Chen, C. A. , Bujor, A. M. , Trojanowski, M. A. , Gopal, D. M. , LaValley, M. P. , Soylemez Wiener, R. , & Klings, E. S. (2021). The prognostic value of cardiac axis deviation in systemic sclerosis‐related pulmonary hypertension. Arthritis Care Res (Hoboken), 74(7), 1219–1226. https://doi.10.1002/acr.24724 10.1002/acr.24724 PMC8639831 34085410

[brb32991-bib-0024] Luoto, R. , Manolio, T. , Meilahn, E. , Bhadelia, R. , Furberg, C. , Cooper, L. , & Kraut, M. (2000). Estrogen replacement therapy and MRI‐demonstrated cerebral infarcts, white matter changes, and brain atrophy in older women: The cardiovascular health study. Journal of the American Geriatrics Society, 48, 467–472. 10.1111/j.1532-5415.2000.tb04990.x 10811537

[brb32991-bib-0025] Merkler, A. E. , Bartz, T. M. , Kamel, H. , Soliman, E. Z. , Howard, V. , Psaty, B. M. , Okin, P. M. , Safford, M. M. , Elkind, M. S. V. , & Longstreth, W. T. (2021). Silent myocardial infarction and subsequent ischemic stroke in the cardiovascular health study. Neurology, 97, e436–e443. https://doi.10.1212/WNL.0000000000012249 34031202 10.1212/WNL.0000000000012249 PMC8356380

[brb32991-bib-0026] Merkler, A. E. , Sigurdsson, S. , Eiriksdottir, G. , Safford, M. M. , Phillips, C. L. , Iadecola, C. , Gudnason, V. , Weinsaft, J. W. , Kamel, H. , Arai, A. E. , & Launer, L. J. (2019). Association between unrecognized myocardial infarction and cerebral infarction on magnetic resonance imaging. JAMA neurology, 76, 956–961. https://doi.10.1001/jamaneurol.2019.1226 31107514 10.1001/jamaneurol.2019.1226 PMC6537766

[brb32991-bib-0027] Nakanishi, K. , Jin, Z. , Homma, S. , Elkind, M. S. V. , Rundek, T. , Tugcu, A. , Yoshita, M. , Decarli, C. , Wright, C. B. , Sacco, R. L. , & Di Tullio, M. R. (2017). Left ventricular mass‐geometry and silent cerebrovascular disease: The cardiovascular abnormalities and brain lesions (CABL) study. American Heart Journal, 185, 85–92. https://doi.10.1016/j.ahj.2016.11.010 28267479 10.1016/j.ahj.2016.11.010 PMC5341701

[brb32991-bib-0028] Rosano, C. , Kuller, L. H. , Chung, H. J. , Arnold, A. M. , Longstreth, W. T. , & Newman, A. B. (2005). Subclinical brain magnetic resonance imaging abnormalities predict physical functional decline in high‐functioning older adults. Journal of the American Geriatrics Society, 53, 649–654. 10.1111/j.1532-5415.2005.53214.x 15817012

[brb32991-bib-0029] Sawano, M. , Yuan, Y. a. , Kohsaka, S. , Inohara, T. , Suzuki, T. , Okamura, T. , Howard, G. , Howard, V. J. , Judd, S. , Soliman, E. Z. , & Cushman, M. (2020). Electrocardiographic ST‐T abnormities are associated with stroke risk in the REGARDS study. Stroke; A Journal of Cerebral Circulation, 51, 1100–1106. https://doi.10.1161/STROKEAHA.119.028069 10.1161/STROKEAHA.119.028069 PMC7122793 32126939

[brb32991-bib-0030] Silva, M. ‐R. A. , Palhares, D. , Ribeiro, L. , Gomes, P. , Macfarlane, P. , Ribeiro, A. , & Marcolino, M. (2021). Prevalence of major and minor electrocardiographic abnormalities in one million primary care Latinos. Journal of Electrocardiology, 64, 36–41. https://doi.10.1016/j.jelectrocard.2020.11.013 33310477 10.1016/j.jelectrocard.2020.11.013

[brb32991-bib-0031] Wardlaw, J. M. , Smith, C. , & Dichgans, M. (2013). Mechanisms of sporadic cerebral small vessel disease: Insights from neuroimaging. The Lancet Neurology, 12, 483–497. 10.1016/s1474-4422(13)70060-7 23602162 PMC3836247

[brb32991-bib-0032] Yaghi, S. , Bartz, T. M. , Kronmal, R. , Kamel, H. , Gottdiener, J. , Longstreth, W. T. , & Elkind, M. S. V. (2018). Left atrial diameter and vascular brain injury on MRI: The cardiovascular health study. Neurology, 91, e1237–e1244. https://doi.10.1212/WNL.0000000000006228 30158157 10.1212/WNL.0000000000006228 PMC6161543

