# Supplementary material for: Electrocardiogram properties and risk of covert brain infarction and other magnetic resonance imaging abnormalities in a stroke‐free population
Source: Brain Behav. 2023 Apr 16;13(5):e2991. doi: 10.1002/brb3.2991 (PMC10175980; doi:10.1002/brb3.2991)
Supplement: Supplementary file 1 — Supplementary material [file BRB3-13-e2991-s002.docx]

Supplemental Table 1 Relationship between ECG abnormalities and covert brain infarcts after excluding hypertensive and atrial fibrillation participants

| Primary outcomes | incident covert brain infarcts | |
| --- | --- | --- |
|  | n=85/516 |  |
|  | OR (95% CI) | *p* |
| **Major ECG abnormalities before 1st MRI scan** |  |  |
| First degree atrio-ventricular block | 1.05（0.34-3.27） | 0.927 |
| Left ventricular hypertrophy | 1.20（0.09-16.01） | 0.896 |
| Ventricular conduction defect | 2.43（0.70-8.47） | 0.162 |
| Major Q-wave abnormalities | 0.77（0.20-3.01） | 0.705 |
| Isolated ST-T wave abnormalities | 0.53（0.11-2.62） | 0.435 |
| **Minor ECG abnormalities before 1st MRI scan** |  |  |
| Minor Q, QS waves | 0.35（0.06-1.89） | 0.221 |
| High R waves | 1.00（0.20-4.96) | 0.995 |
| Minor isolated ST-T abnormalities | 1.05（0.39-2.83） | 0.927 |
| ST elevation* | - | 0.994 |
| Incomplete RBBB | 2.48（0.81-7.62） | 0.113 |
| Long QT interval | 0.50（0.13-1.89） | 0.307 |
| Short PR | 1.52（0.16-14.68） | 0.718 |
| Left axis deviation | 1.79（1.04-3.10） | 0.037 |
| Right axis deviation | 0.24（0.03-2.31) | 0.218 |
| Minor Q, QS waves with ST-T abnormalities | 6.32（0.84-47.41) | 0.073 |

*The number of participants with ST elevation was 4, so the confidence interval was too wide to show it. Models adjusted for age, sex, race, body mass index, systolic blood pressure, antihypertensive drug therapy, smoking status, diabetes mellitus, congestive heart failure, myocardial infarction, left atrial dimension, and all ECG-measured parameters.
