# Supplementary material for: Electrocardiogram properties and risk of covert brain infarction and other magnetic resonance imaging abnormalities in a stroke‐free population
Source: Brain Behav. 2023 Apr 16;13(5):e2991. doi: 10.1002/brb3.2991 (PMC10175980; doi:10.1002/brb3.2991)
Supplement: Supplementary file 2 — Supplementary material [file BRB3-13-e2991-s001.docx]

Supplemental Table 2 Relationship between ECG abnormalities and non-lacunar ischemic stroke occurrence

| Primary outcomes | incident non-lacunar ischemic stroke | |
| --- | --- | --- |
|  | n=255/3965 |  |
|  | OR (95% CI) | *p* |
| **Major ECG abnormalities before 1st MRI scan** |  |  |
| First degree atrio-ventricular block | 1.13（0.72-1.77） | 0.603 |
| Left ventricular hypertrophy | 0.83（0.45-1.54） | 0.553 |
| Ventricular conduction defect | 1.46（0.92-2.31） | 0.108 |
| Major Q-wave abnormalities | 1.41（0.88-2.27） | 0.158 |
| Isolated ST-T wave abnormalities | 1.27（0.77-2.10） | 0.352 |
| **Minor ECG abnormalities before 1st MRI scan** |  |  |
| Minor Q, QS waves | 1.07（0.56-2.04） | 0.839 |
| High R waves | 1.35（0.77-2.36） | 0.293 |
| Minor isolated ST-T abnormalities | 1.51（1.03-2.21） | 0.036 |
| ST elevation | 0.34（0.05-2.63） | 0.304 |
| Incomplete RBBB | 0.44（0.16-1.22） | 0.116 |
| Long QT interval | 1.00（0.67-1.51） | 0.986 |
| Short PR | 1.97（0.86-4.48） | 0.107 |
| Left axis deviation | 1.05（0.79-1.41） | 0.741 |
| Right axis deviation | 0.81（0.37-1.79） | 0.606 |
| Minor Q, QS waves with ST-T abnormalities | 1.23（0.54-2.82） | 0.619 |

Models adjusted for age, sex, race, body mass index, systolic blood pressure, antihypertensive drug therapy, smoking status, diabetes mellitus, congestive heart failure, myocardial infarction, atrial fibrillation, left atrial dimension, and all ECG-measured parameters.
